# The production of a recombinant tandem single chain fragment variable capable of binding prolamins triggering celiac disease

**DOI:** 10.1186/s12896-018-0443-0

**Published:** 2018-05-29

**Authors:** Britta Eggenreich, Elke Scholz, David Johannes Wurm, Florian Forster, Oliver Spadiut

**Affiliations:** 1 Research Division Biochemical Engineering, Institute of Chemical, Environmental and Bioscience Engineering, TU Wien, Vienna, Austria; 2 Sciotec Diagnostics Technologies GmbH, Ziegelfeldstr. 3, 3430 Tulln, Austria

**Keywords:** Celiac disease, Single chain fragment variable, *E. coli*, Inclusion body, ELISA

## Abstract

**Background:**

Celiac disease (CD) is one of the most common food-related chronic disorders. It is mediated by the dietary consumption of prolamins, which are storage proteins of different grains. So far, no therapy exists and patients are bound to maintain a lifelong diet to avoid symptoms and long-term complications. To support those patients we developed a tandem single chain Fragment variable (tscFv) acting as a neutralizing agent against prolamins. We recombinantly produced this molecule in *E. coli*, but mainly obtained misfolded product aggregates, so-called inclusion bodies, independent of the cultivation strategy we applied.

**Results:**

In this study, we introduce this novel tscFv against CD and present our strategy of obtaining active product from inclusion bodies. The refolded tscFv shows binding capabilities towards all tested CD-triggering grains. Compared to a standard polyclonal anti-PT-gliadin-IgY, the tscFv displays a slightly reduced affinity towards digested gliadin, but an additional affinity towards prolamins of barley.

**Conclusion:**

The high binding specificity of tscFv towards prolamin-containing grains makes this novel molecule a valuable candidate to support patients suffering from CD in the future.

**Electronic supplementary material:**

The online version of this article (10.1186/s12896-018-0443-0) contains supplementary material, which is available to authorized users.

## Background

Celiac disease (CD) is one of the most common food-related chronic disorders with a prevalence of 1–2% in Western nations [1, 2]. It is triggered by the dietary consumption of storage proteins (prolamin, alcohol soluble fraction of gluten) of wheat, barley, rye and others [3, 4]. Up to date it is still not completely clear which factors lead to the manifestation of CD. Genetically, patients carry genes for the human leukocyte antigens HLA-DQ2 and HLA-DQ8, but also environmental factors, like early exposure to dietary gluten, infection and/or change in the bacterial flora of the intestine contribute to this disorder [1, 3–5].

**Fig. 1:**
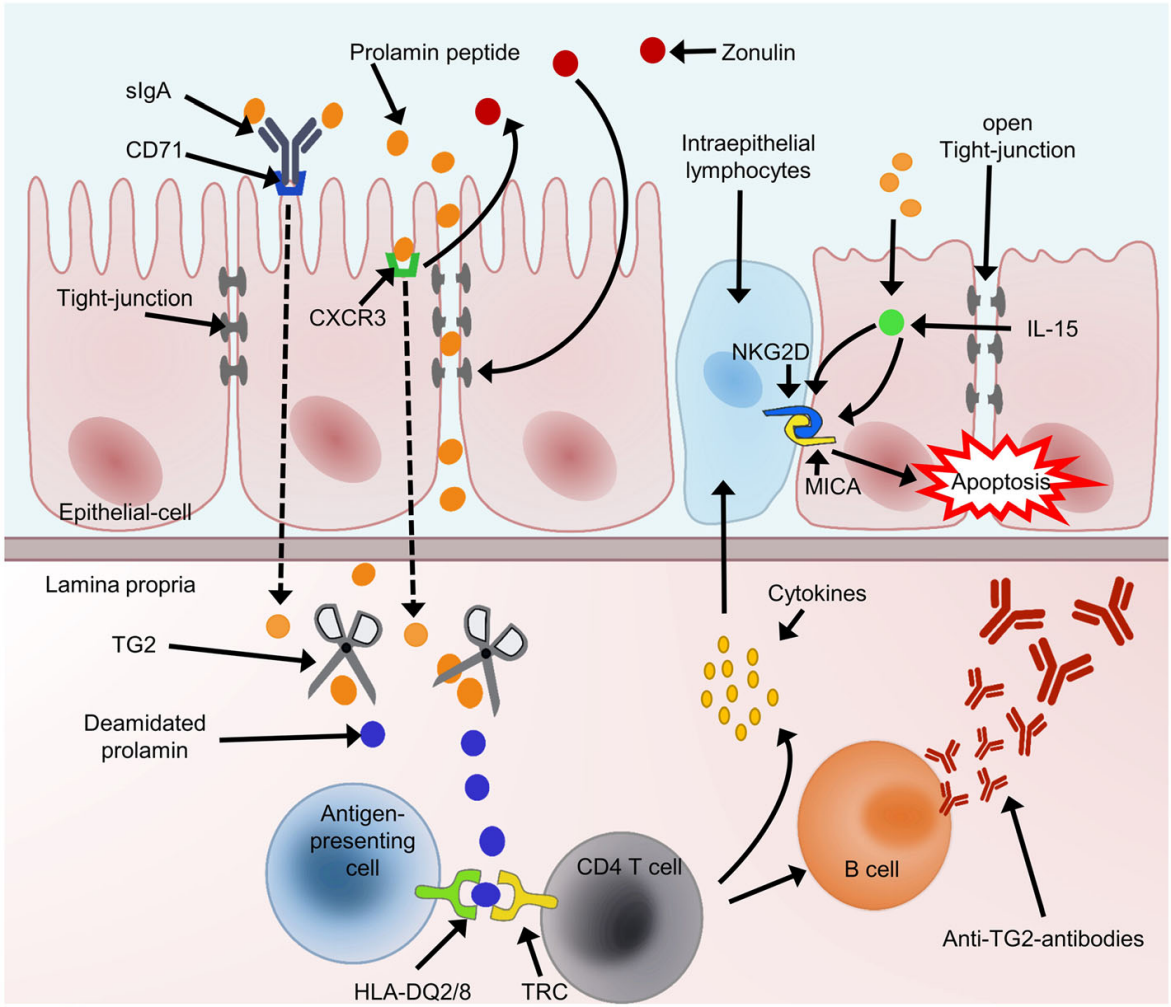
Adapted simplified pathogenesis of celiac disease [3, 5, 9]. Prolamin overcomes the epithelial barrier via a transcellular transport as a soluble IgA-prolamin complex bound to an epithelial receptor (CD71). The interaction of prolamin with a chemokine receptor CXCR3 leads to the release of Zonulin, a protein that increases the permeability of the epithelium, due to opening of Tight-junctions and hence allows paracellular transport of prolamin. CD71, CXCR3 and Zonulin are upregulated in patients with celiac disease. Prolamin that reaches the lamina propria gets deamidated by transglutaminase 2 (TG2) and hence binds more strongly to human leukocyte antigens (HLA)-DQ2 and DQ8 molecules on antigen-presenting cells. These presented prolamins activate CD4^+^T-cells, which then secrete proinflammatory cytokines. Furthermore, T-cells induce the expression of Interleukin (IL) 15 and autoantibodies against TG2 by innate immune cells. IL 15 has a very important role regarding the remodeling process of the intestinal surface. It leads to an upregulation of nonconventional HLA molecules, MICA on enterocytes, and activates NKG2D receptors on intraepithelial lymphocytes (IELs). The interaction of MICA and NKG2D promotes the downstream effect of IEL-mediated epithelial damage. Another source of IL 15 are epithelial and dendritic cells after contact with prolamin. To sum up, the contact of prolamin with the epithelial layer activates the innate and humoral immune system, which induces the destruction of the surface of the small intestine

In patients with CD the uptake of gluten leads to the secretion of autoantibodies and tissue transglutaminase (TG2), as well as proinflammatory cytokines, such as Interleukin (IL) 15, IL 21, Tumor Necrosis Factor (TNF) alpha and Interferon (IFN) gamma (Fig. 1) [1, 3]. Thus, inflammations of the small bowel occur, ranging from intraepithelial lymphocytosis up to total villous atrophy combined with crypt hyperplasia [1, 3]. Hence, symptoms vary between asymptomatic, extra-intestinal manifestations, various abdominal complications, up to global malabsorption [3, 6]. Long-term complications include malignancy, such as intestinal lymphomas and adenocarcinoma [3, 7, 8].

To reduce symptoms and avoid long-term complications, a strict gluten free diet (GFD) is the only effective treatment of CD so far [3]. Due to the high prevalence, severe symptoms, long-term complications and limited treatment possibilities, it is self-explanatory that patients are in pressing need of additional and alternative therapies. Many novel drugs are in development and the results of the respective clinical trials are impatiently anticipated. As shown in Table 1 various novel therapies are under development, however none of these has reached clinical phase 3 investigations yet. Hence, unfortunately no novel therapy will be introduced to the market in the near future. Next to this lack of therapeutic options, a high social burden lies upon patients with CD, because a lifelong GFD is difficult to maintain. Even in “gluten-free” dietary products traces of prolamins are found, which have a severe impact on the well-being [10]. To support those patients we recently developed a novel single chain Fragment variable (scFv) against prolamins [11]. This scFv works as a “neutralizing agent”, meaning that a complex between prolamin and the scFv is formed in the gut and no systemic interactions are expected, as the formed complex does not cross the epithelial barrier and is finally excreted. Thus, the scFv can be applied as a medical device. To obtain this novel scFv, we immunized chicken with peptic tryptic digested gliadin (PT-gliadin). Those immunized chicken were used as source for RNA, carrying the sequence for the recombinant scFv [11]. Since no effector function of the antibody (AB) is relevant for the neutralizing effect, but only the variable light and heavy chain are required, we generated a single chain Fragment variable (scFv). Since two antigen binding regions increase binding affinity, we joined two scFv with a peptide linker and constructed a tandem single chain Fragment variable (tscFv) [12, 13]. A block flow diagram of this process is presented in Additional file 1: Figure S1.

**Table 1:** Potential therapies/supplementations for patients with celiac disease

Site of action	Target	Principle of effect	Information/Drug	Phase of clinical trial	ClinicalTrials.gov Identifier	Ref.
Intra-luminal	Flours	Pretreatment with lactobacilli, transamidation of gliadin	Microbial Transglutaminase and Lysine Ethyl Ester (WHETMIT)	Phase 2	NCT02472119	[5]
	Prolamin	Polymetric binders, form high affinity complexes with alpha-gliadin	Poly-hydroxyethylmethacrylate-co-styrene sulfonate BL-7010	Phase 2	NCT01990885	[5]
	Prolamin	Antibodies or Antibody-fragments with high affinity to prolamin ➔ neutralizing effect	Tandem single chain Fragment variable directed against prolamins of different grains (Glutosin ™)			[10]
	Prolamin	Peptidase based, enzymes to degrade prolamin	• Cystein-Endopeptidas B2, Prolin-Endopeptidase (ALV003), • Cocktail of microbial enzymes (STAN 1) • Prolyl endopeptidase (AN-PEP)	Phase 1 + 2 Phase 1 + 2 Phase 1 + 2	NCT01255696 NCT00962182 NCT00810654	[5, 11]
	Prolamin	Bifidobacteria and lactobacillus species that hydrolyse gliadin	*Bifidobacteria infantis* and lactobacillus species		NCT01257620	[5]
	Prolamin	Desensitizing	*Necator americanus* • (NaCeD) • (NainCeD-3)	Phase 1 + 2 Phase 1	NCT01661933 NCT02754609	[5]
Epithelial layer	Zonulin receptors	Antagonizing Zonulin recetors, tight junction modulation	Larazotide acetate (AT-1001)	Phase 2	NCT01396213	[5, 12]
	Transcellular gliadin transport	Inhibition of sIgA-CD71 mediated transport				[5]
	IL 15	IL 15 action is blocked	• Humanized Mik-Beta-1 Monoclonal Antibody Directed Toward IL-2/IL-15R Beta (CD122) (Hu-Mik- Beta-1) • Human monoclonal antibody (AMG 714)	Phase 1 Phase 2	NCT01893775 NCT02637141	[12]
Lamina propria	HLA- DQ2 or DQ8	Blocking HLA-DQ2 or DQ8				[12]
	CCR3	CCR3 blocking to repress T cell homing	CCX282-B		NCT00540657	[13]
	TG2	Inhibition of TG2				[12]
	Cathepsin-S inhibitor	Participate in the degradation of antigenic proteins to peptides for presentation on MHC class II	RG7625	Phase 1	NCT02679014	[14]
Immune system	Immune response	Vaccination	Nexvax2	Phase 1	NCT02528799	[12]

**Fig. 2:**
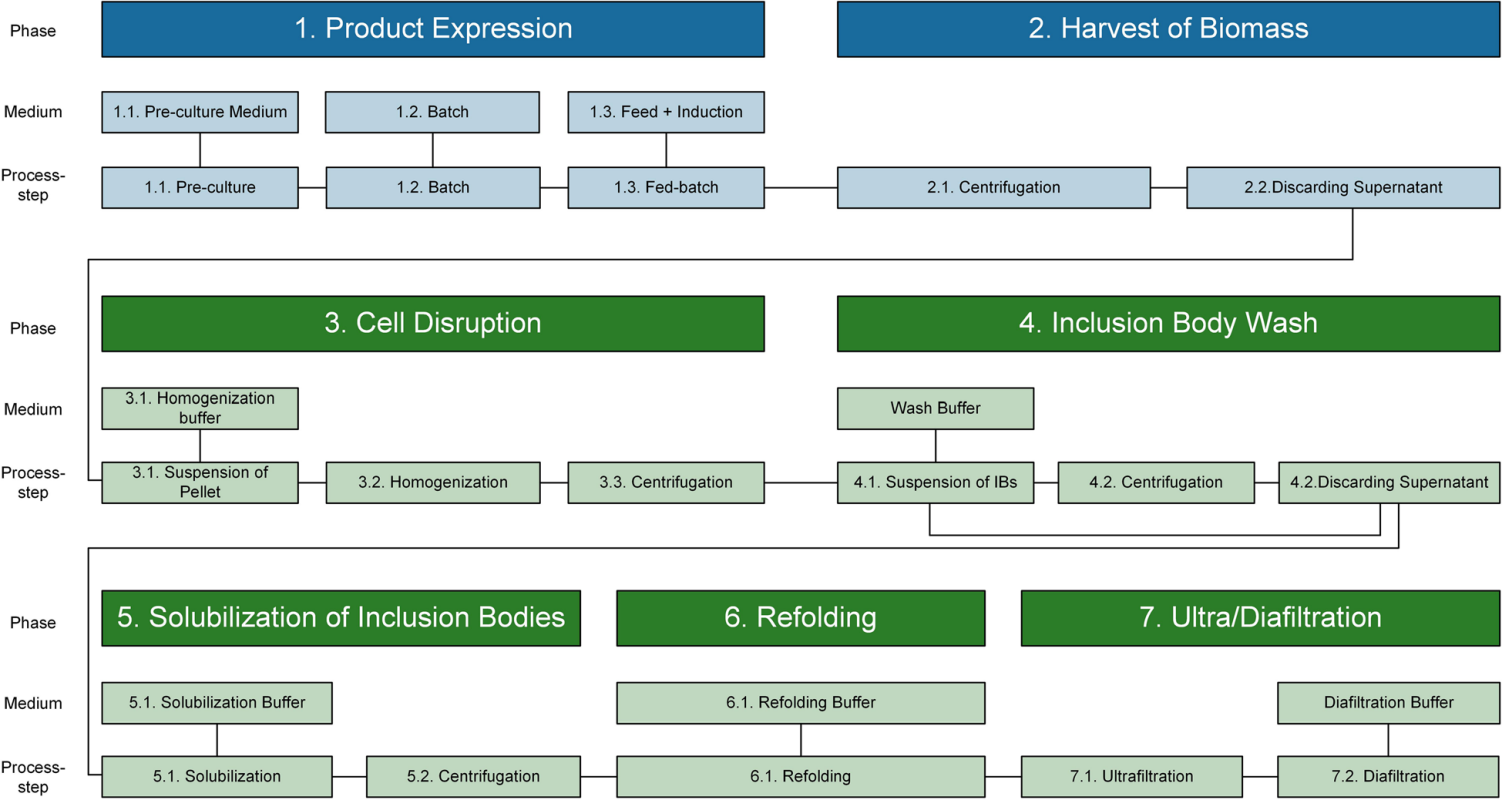
A typical Up- (in blue) and Downstream (in green) for Inclusion Body processing

We selected *Escherichia coli* as production organism for recombinant tscFv, since *E.coli* is a common host for scFv production, due to its advantages of high cell density cultivations and high product titers [14–16]. Nevertheless, high translational rates, strong promotor systems and intrinsic product features often result in the formation of insoluble product aggregates, so-called Inclusion Bodies (IB) [17]. Downstream processing (DSP) of IBs is laborious and contains several steps including at least IB recovery, solubilization and refolding as key unit operations [17, 18]. A typical IB process is schematically shown in Fig. 2.

Besides the complexity of an IB process, the commonly low refolding yields describe further challenges [18–20]. On the other hand, IBs describe an efficient production strategy, not only because more than 30% of the cellular protein can be produced as IBs, but also because IBs contain a high level of the recombinant product, which is protected against proteolysis [18, 21].

In the current study, we recombinantly produced the novel tscFv in *E. coli* as IBs, processed the IBs following a standardized protocol and characterized the refolded product. Summarizing, we introduce a novel, recombinant tscFv as an interesting biological agent to treat patients with CD.

## Methods

### Chemicals

All chemicals were purchased from Carl Roth GmbH (Vienna, Austria), if not stated otherwise.

### Strains and tscFv production

#### Strain and construct

The gene coding for the tandem single chain fragment variable (tscFv) against PT-gliadin was cloned into the pET-28a(+) vector with an additional stop codon upstream of the his_6_-tag. Subsequently, the plasmid was transformed into *E.coli* BL21(DE3) [11].

#### Bioreactor cultivations

Bioreactor cultivations were performed according to our previous study [22]. In short, 500 mL pre-culture (DeLisa medium [23]; 50 μg/mL Kanamycin) were used to inoculate 4500 mL sterile DeLisa medium in a stainless steel Sartorius Biostat Cplus bioreactor (Sartorius, Göttingen, Germany) with a working volume of 10 L. After a batch (maximum specific growth rate (μ_max_): 0.6 h^− 1^; biomass end of batch: 8.1 g dry cell weight/L (DCW/L)) and a non-induced fed-batch (μ: 0.09 h^− 1^; biomass end of non-induced fed-batch: 47.6 g DCW/L) for biomass (BM) generation, cells were induced with 0.5 mM Isopropyl β-D-1-thiogalactopyranoside (IPTG) at 30 °C for 10 h (μ: 0.05 h^− 1^; biomass end of induced fed-batch: 56.2 g DCW/L). Throughout the whole cultivation pH was kept at 7.2 and dissolved oxygen above 40%. Biomass was harvested by centrifugation (179 g, 20 min, 4 °C) and stored at − 20 °C.

##### Sampling strategy

Samples were taken at the beginning and the end of the batch, non-induced fed-batch and induced fed-batch. Specific product formation rates and final product yields were calculated for the induction phase of approximately 10 h. Dry cell weight (DCW) was determined in triplicates, by centrifugation (21,913 g, 4 °C, 10 min) of 1 mL cultivation broth, washing the obtained cell pellet with a 0.1% NaCl solution and subsequent drying at 105 °C for 48 h. Product, substrate and metabolites were quantified as described in our previous study [22].

### IB processing

#### IB recovery and purification

Prior to cell disruption, frozen BM was thawed at 4 °C and suspended in 50 mM Tris-HCl buffer, pH 8.0. BM concentration was adjusted to 10 g DCW/L. Cell disruption was performed by high-pressure homogenization using a PandaPLUS 2000 (GEA Mechanical Equipment, Parma, Italia). In total, 3 passages at 1500 bar were used to disrupt the cells. These conditions were chosen based on our previous study [24]. To limit heat generation, BM was kept on ice and a cooling unit was connected to the outlet of the homogenizer. Disrupted BM was centrifuged (15,650 g, 4 °C, 20 min) and the supernatant was discarded. Then, IBs were washed with deionized water (100 g wet weight/L (WW/L)). To ensure a homogeneous mixture, a T10 basic ULTRA-TURRAX® (IKA, Staufen, Germany) was used (2 min, stage 5, 4 °C). The suspension was centrifuged (15,650 g, 4 °C, 20 min) and the supernatant was discarded. This wash procedure was performed twice.

#### IB solubilization and refolding

100 g WW/L of washed IBs were resuspended in solubilization buffer (50 mM TRIS, 2 M Urea, 10% *v*/v Glycerol, pH 12; [18]). The suspension was kept in an Infors HR Multitron shaker (Infors, Bottmingen, Switzerland) at room temperature (RT) at 100 rpm. After 60 min, the solution was centrifuged (15,650 g, 4 °C, 20 min) to remove insoluble cell components.

Refolding was performed by dilution. Solubilized IBs were added to the refolding buffer (50 mM Tris-HCl, 2 M Urea, 10% v/v Glycerol, pH 8.5, adjusted from [25, 26]) to reach a protein concentration of 0.5 mg/mL, corresponding to a 50-fold dilution. The refolding preparation was kept at 14 °C and 100 rpm in an Infors HR Multitron shaker (Infors, Bottmingen, Switzerland) for 48 h. Yields were calculated based on HPLC measurements (see section “*HPLC measurement**”*).

#### Ultra- and diafiltration

Re-buffering (50 mM Tris-HCl, 5% *w*/*v* Mannitol, pH 8.0) and concentration was performed with a Centramate™ 500 S Tangential Flow Filtration System (Pall, Austria; Vienna). Due to the calculated size of the tscFv of 52.9 kD, a Centramate Cassette with a 10 kD cutoff and 0.1 m^2^ filtration area was used. Transmembrane pressure was kept below 0.7 bar. Prior to storage at − 20 °C, product aggregates were removed by filtration (0.2 μm pore-size).

### Biological assays

#### Enzyme-linked immunosorbent assay (ELISA)

To reassure the ability of the refolded product to neutralize antigens, ELISA analyses were performed. 96 well ELISA plates were either coated with 100 ng/well PT-gliadin or coated with 1% *w*/*v* PEG 6000 as negative control. We described the coating protocol as well as the ELISA in detail in our previous study [11]. To reduce unspecific interactions, samples containing refolded tscFv or tscFv IBs were diluted with Tris-buffered saline (24.8 mM Tris, 136.9 mM NaCl and 2.7 mM KCl, pH 8.0) containing 0.05% Tween 20 (TBST). 100 μL sample/well were incubated for an hour at 25 °C and 450 rpm. Every well was washed three times with 300 μL TBST. Subsequently, 100 μL of a 1:1000 dilution of Anti-Chicken IgG (H + L), F(ab′)2 fragment-Peroxidase antibody produced in rabbit (Sigma, Vienna, Austria) with TBST were added per well and incubated at 37 °C and 450 rpm for an hour (THERMOstar microplate incubator, BMG Labtech, Ortenberg, Germany). Each well was washed four times with 300 μL TBST. A color reaction was mediated by the addition of 100 μL premixed 3,3′,5,5′-tetramethylbenzidine (TMB) substrate (Thermo Scientific, Vienna, Austria), which reacted with the peroxidase. After 15 min, 50 μL of 0.9 M HCl were added as stop reagent. Absorbance was measured at 450 nm in a Multiskan FC Microplate Photometer (Thermo Scientific, Vienna, Austria).

#### Competitive ELISA

To determine the binding affinity of the refolded product to a variety of prolamins of different flours, competitive ELISAs were performed. For this purpose, flours of different plants were digested with simulated gastric fluid (0.1 mM pepsin from porcine gastric mucosa, 55 mM NaCl, pH 1.2) at 37 °C for 1 h. The digest was centrifuged (2647 g, 5 min) and the pH of the supernatant was adjusted to 8.5. Precipitating proteins were removed by centrifugation (2647 g, 5 min) and the protein content of the supernatant was determined. Different concentrations (1000, 500, 250, 125, 75, 0.01 and 0.0 μg total protein/mL) of these digested flours (rye, barley, buckwheat, rice, maize, kamut, almond, soy, millet, spelt and wheat) were added to the ELISA plate with sample (refolded tscFv, tscFv IBs) and TBST, incubated and developed as described in *2.4.1*. Due to this setup the applied digested flours and the immobilized PT-gliadin were competing over tscFv. Samples, which bound to predigested flours in the supernatant were washed away and thus the absorption signal was reduced. As positive control, anti-PT-gliadin-IgY extracted from egg yolk of PT-gliadin immunized hens was used. Also, a standard competitive ELISA, where PT-gliadin was competing against itself, was included.

##### Half maximal inhibitory concentration (IC50)

IC50 values were calculated to exemplify competitive ELISA results. The values show the total protein concentration of predigested grains, which is necessary to reduce the detectable signal by half. Low IC50 values indicate a high affinity to the flours in the supernatant. IC50 values were calculated using SigmaPlot (Systat Software, San Jose, USA). A non-linear regression was performed and the equation for standard and four parameter logistic curves was used (Eq. 1).

1 $$ y=\mathit{\min}+\frac{\left(\mathit{\max}-\min \right)}{1+{\left(x/ IC50\right)}^{- Hillslope}} $$

, where min is the bottom and max the top of the curve. Hillslope stands for the slope of the curve at its midpoint.

### Analytics

#### Protein measurement

The protein content was determined using Bradford Coomassie Blue assay or Bicinchoninic acid assay (Sigma-Aldrich, Vienna, Austria). Bovine serum albumin (BSA) was used as a standard. To stay in the linear range of the detector (Genesys 20, Thermo Scientific, Waltham, MA, USA) samples were diluted with the respective buffer.

#### HPLC measurement

HPLC measurements were performed to gain information about 1) the purity of the solubilized IBs and 2) the purity and content of correctly refolded product. Therefore, particle-free samples of 5 μl were analyzed by an UltiMate™ 3000 HPLC with a MAbPac™ SEC-1 size exclusion column and an UltiMate™ 3000 Multiple Wavelength Detector (Thermo Scientific, Vienna, Austria). The mobile phase was either a 50 mM BisTris buffer containing 4 M Guanidinhydrochlorid (GnHCl) and 100 mM NaCl (pH 6.8) for solubilized IBs, or 100 mM NaH_2_PO_4_ buffer containing 300 mM NaCl (pH 6.8) for the refolded product, respectively. The system was run with an isocratic flow of 100 μl/min at 25 °C column oven temperature. Every HPLC run included measurements of 29 kD, 43 kD and 75 kD size standards (Gel Filtration LMW Calibration Kit, GE Healthcare, Vienna, Austria). Recorded chromatographic data at 280 nm were analyzed using OriginPro 9.1 (OriginLab Corporation, Northampton, United States). Since baseline separation was not achieved, borders (points of inflection) for peak integration were obtained by calculating the first derivative of the chromatographic data. Refolding yields were calculated using Eqs. 2–5. Areas of Standard proteins differed depending on the used mobile phase: using GnHCl-containing buffer the area was smaller by a factor of 1.195 ± 0.0027. Hence, this factor was used as a correction factor during yield calculations.

2 $$ AUC\; total\; sol\; target=\frac{AUC\; sol\; target}{injection\kern0.17em volume}\ast volume\; sol $$

3 $$ AUC\; corr\kern0.17em total\; sol= AUC\; total\; sol\; target\ast 1.195 $$

4 $$ AUC\; expected\ target=\frac{Area\kern0.17em corr\kern0.17em total\; sol}{volume\; end}\ast injection\kern0.17em volume $$

5 $$ Yield=\frac{AUC\; measured\kern0.17em target}{AUC\; expected\kern0.17em target}\ast 100 $$

#### Product identification/qualification

Product and host cell impurities in refolded product were analyzed by SDS-Page and subsequent mass spectrometry (MS) analysis. Therefore, bands of interest were excised from the gel, samples were digested with Trypsin (Promega, Mannheim, Germany) and proteins were S-alkylated with iodoacetamide. Peptides were extracted from the gel by a couple of washing steps. The digested samples were loaded on a BioBasic-18, 150 × 0.32 mm, 5 μm column (Thermo Scientific, Vienna, Austria) using 65 mM Ammonium formate buffer (buffer A) as aqueous solvent. A gradient from 5% B (B: 100% Acetonitrile) to 32% B in 45 min was applied, followed by a 15 min gradient from 32% B to 75% B that facilitated elution of large peptides at a flow rate of 6 μL/min. Detection was performed with MaXis 4G Q-TOF-MS (Bruker,Billerica MA, USA) equipped with the standard Electrospray ionization (ESI) source in positive ion, DDA mode (= switching to MSMS mode for eluting peaks). MS-scans were recorded (range: 150–2200 Da) and the six highest peaks were selected for fragmentation. Instrument calibration was performed using ESI calibration mixture (Agilent, Vienna, Austria). Analysis files were converted (using Data Analysis, Bruker) to MGF files, which are suitable for performing a MS/MS ion search with GPM (automated search engine). *E.coli* (strain K12) proteins and product sequence were inserted in the database for sequence identification.

## Results

### Production of tscFv

The fed-batch cultivation yielded 2.3 g IBs per L fermentation broth corresponding to a specific titer of 0.041 g IB/g DCW and a space-time-yield of 0.23 g IB/L/h induction time. The strain-specific physiological parameters are shown in Table 2.

**Table 2:** Strain physiological parameters of *E. coli* BL21(DE3) producing tscFv IBs

	cultivation time	specific glucose uptake rate	growth rate	Biomass concentration	C-balance	specific product titer	volumetric product titer
	[h]	qs Gluc [g/g/h]	μ [h^−1^]	g DCW/L		[mg/g]	[g/L]
Batch	0–6.7	0.62	0.6	8.13	0.95		
Fed-Batch	6.7–22.4	0.29	0.09	47.60	0.89		
Induced Fed-Batch	22.4–32.4	0.20	0.05	56.15	1.01	40.90	2.30

### IB processing

**Fig. 3:**
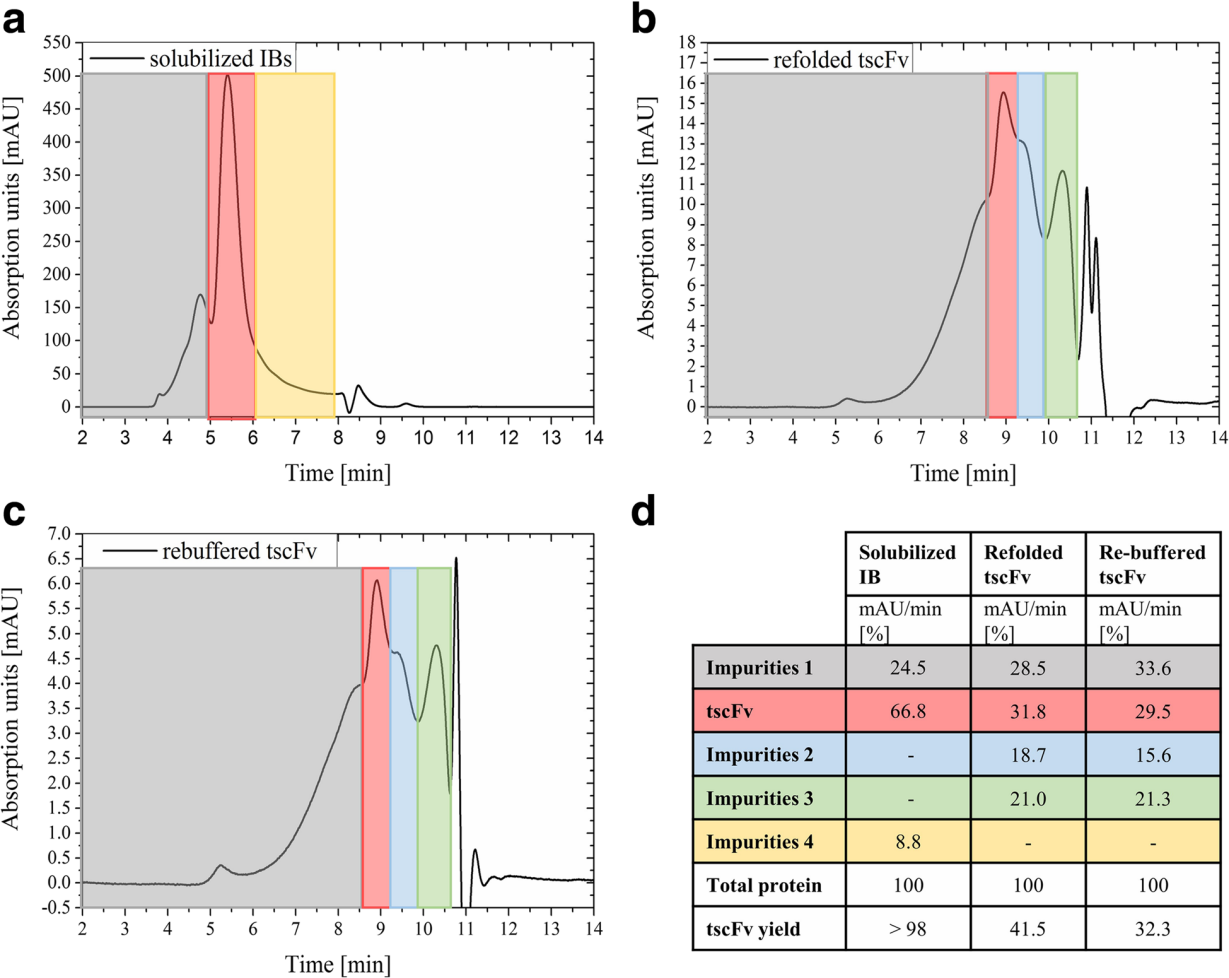
HPLC chromatograms at 280 nm and percentage of protein species. **a**, solubilized IBs; **b**, refolded protein mixture; **c**, refolded product after ultra- and diafiltration; **d**, integral results of the different peaks in percent and yield calculations. Grey, Impurities 1 (lager in size than target protein); red, target protein; blue, Impurities 2; green, Impurities 3; yellow, Impurities 4. The other peaks in the chromatogram are buffer peaks

Buffers and methods for IB processing were either developed in a previous study [24] or adapted from literature [18, 25, 26]. After cell disruption and IB wash, IBs were solubilized followed by refolding. Under the chosen conditions (100 mg WW IB/mL solubilization buffer, solubilized for 1 h at room temperature) approximately 25 mg/mL solubilized protein was found. This mixture of solubilized proteins mainly contained target protein, but also different host cell proteins and other impurities were found (Fig. 3a, d). HPLC measurements of the solubilized IBs revealed a purity of at least 66.8%. This solubilized protein mixture was added to a refolding buffer for 48 h. The refolding yield was calculated with 41.5% target protein (Eqs. 2–5; Fig. 3b, d), prior to concentration and re-buffering. After ultra- and diafiltration, another HPLC measurement was performed. At this step, an increase of impurities smaller than the target protein was found. The resulting chromatogram (Fig. 3c) showed 29.5% correctly folded target protein. Using Eqs. 2–5, the overall refolding yield was calculated with 32.3% (Fig. 3d).

#### MS measurements

**Fig. 4:**
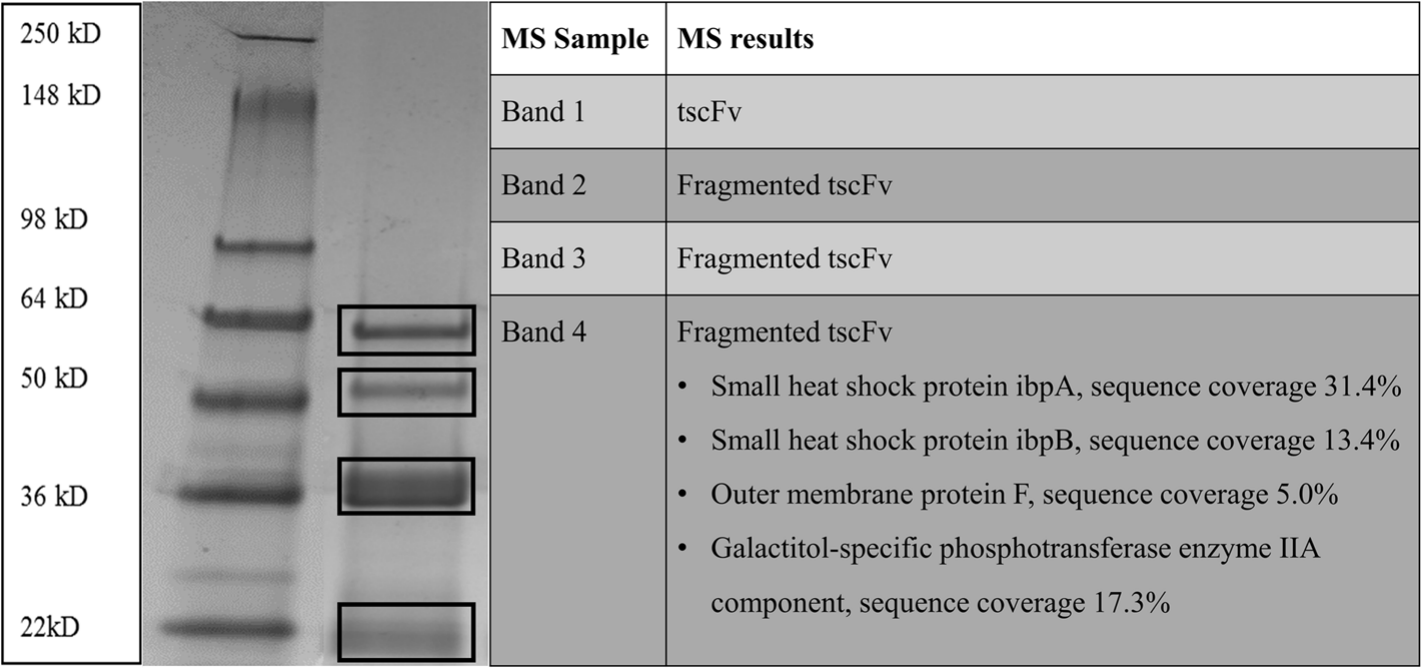
SDS gel for MS analysis and the corresponding results. Left lane represents the protein ladder, right lane the applied refolded tscFv preparation; marked protein bands were excised and analyzed. MS results are presented in the Table. For all host cell impurities percentage of sequence coverage of the MS analysis are given

To investigate the purity of the refolded and diafiltrated tscFv, MS analysis was performed. Therefore, the refolded tscFv was applied on an SDS gel and the different protein bands were excised and analyzed (Fig. 4). The SDS gel showed four dominant protein bands, which all contained the refolded product. Host cell proteins were only found to a small portion in the lowest band, indicating a high purity of the refolded product.

### Biological assays

#### Binding capability of tscFv IBs

**Fig. 5:**
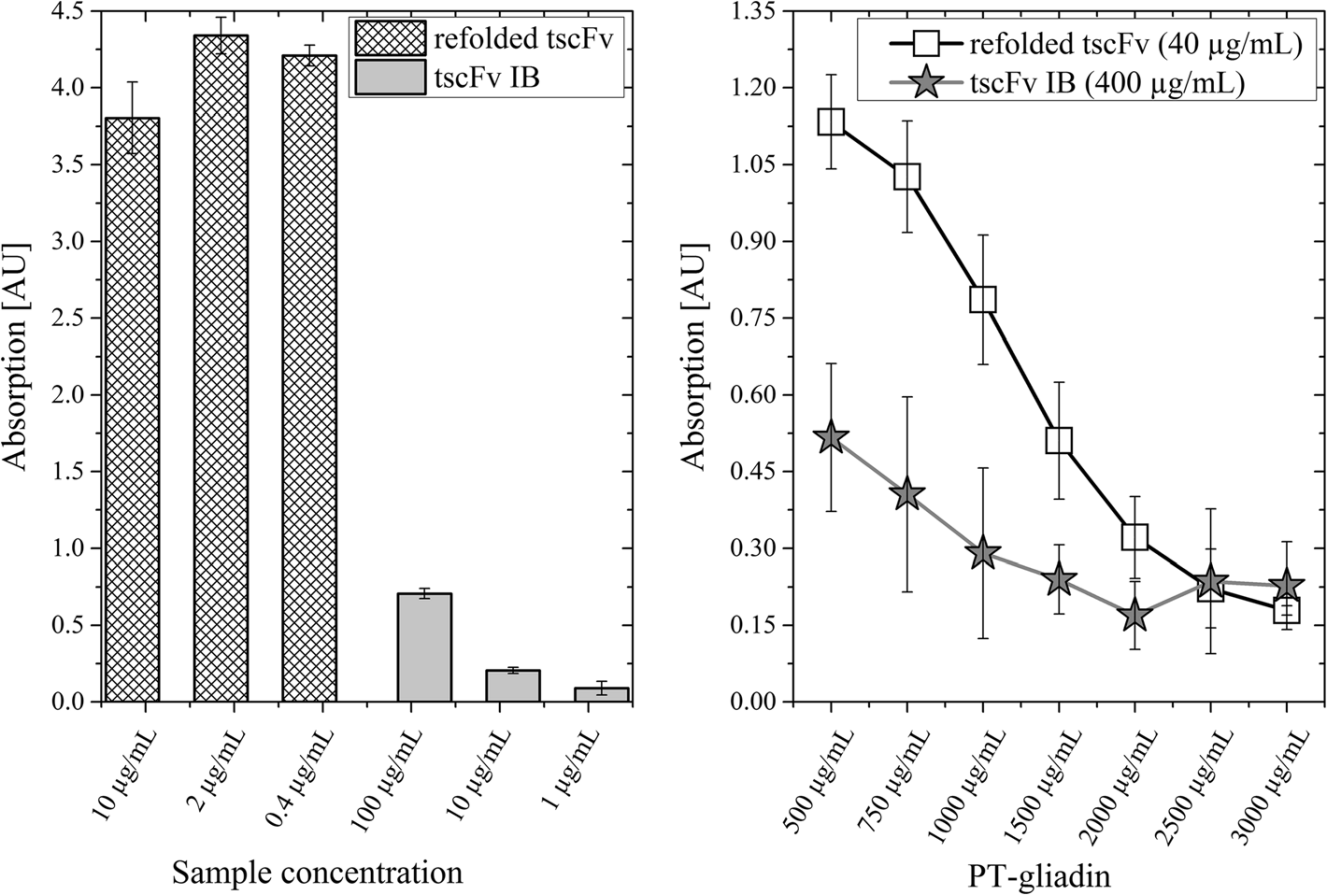
Comparison of the binding capability of refolded tscFv and tscFv inclusion bodies (IBs). A, PT-gliadin ELISA where 10, 2 and 0.4 μg/mL refolded tscFv and 100, 10 or 1 μg/mL lyophilized and resuspended IBs were used; B, competitive ELISA, IBs (400 μg/mL) or refolded tscFv (40 μg/mL) were applied with PT-gliadin and sample buffer. Signal reductions show that the samples are binding to increasing concentrations of PT-gliadin in the supernatant and not to the immobilized PT-gliadin on the plates

Literature has demonstrated that to some extent IBs can exhibit biological activity [27–30]. Therefore, we compared the binding capability of tscFv IBs and refolded tscFv using both a PT-gliadin and a competitive ELISA (Fig. 5). Figure 5a shows a PT-gliadin ELISA with refolded tscFv and tscFv IBs. Low concentrations of refolded tscFv led to no signal reduction of the ELISA, hence even the lowest applied concentration of 0.4 μg/mL saturated the assay. IBs, on the other hand, showed a low signal intensity, meaning that even a 10-times higher concentration of IBs (100 μg/mL) only led to a fifth of the signal intensity compared to refolded tscFv (10 μg/mL). Thus, a much higher IB concentration would be needed to achieve similar results compared to the refolded tscFv. This higher binding capacity of refolded tscFv was also found using a competitive ELISA (Fig. 5, b), where a 10 times higher concentration of IBs was necessary to get comparable results. Summarizing, although tscFv IBs show binding capabilities and do not have to be further processed to capture prolamins, higher concentrations of tscFv IBs are required to lead to the same effect as refolded tscFv.

#### Comparison of refolded tscFv and anti-PT-gliadin-IgY

**Fig. 6:**
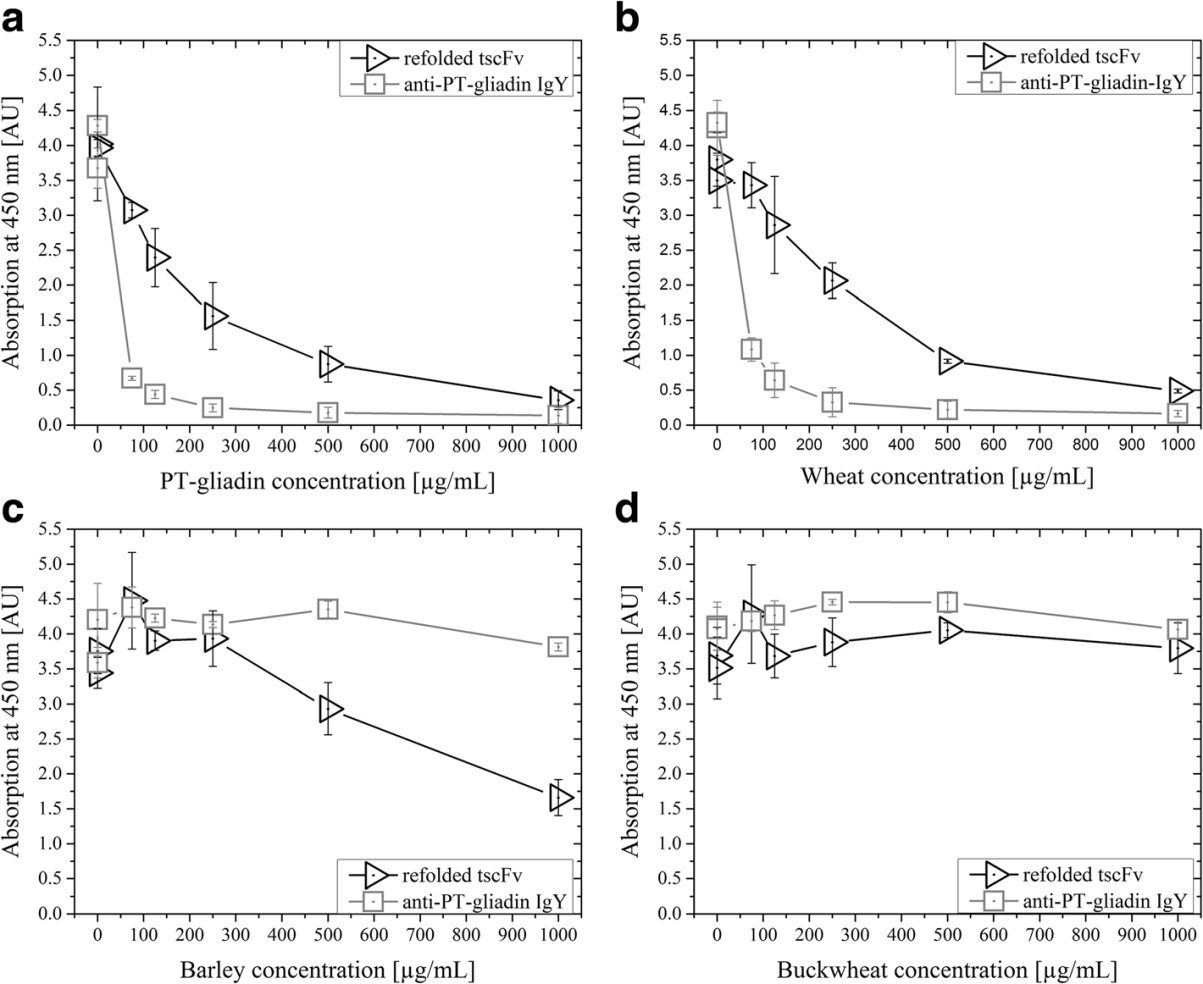
Competitive ELISA of refolded tscFv and anti-PT-gliadin-IgY. 50 μg/ml sample (refolded tscFv or anti-PT-gliadin-IgY) were applied with different concentrations (0, 0.0075, 75, 125, 250, 500 and 1000 μg/mL) of **a**, PT-gliadin; **b**, wheat; **c**, barley; and **d**, buckwheat

In our previous study we showed that soluble scFv and standard anti-PT-gliadin-IgY displayed comparable binding capabilities [11]. In a similar fashion, we tested the refolded tscFv against the model protein PT-gliadin and flour digests of wheat, barley and buckwheat and compared it to anti-PT-gliadin-IgY in a first comparative feasibility experiment (Fig. 6). Wheat is known for its high prolamin content (80% of total proteins; [31]). We chose buckwheat as negative control, due to its reduced prolamin content [32].

As depicted in Fig. 6a and b a reduced concentration of PT-gliadin and digested wheat, respectively, was necessary to replace anti-PT-gliadin-IgY from immobilized PT-gliadin. However, anti-PT-gliadin-IgY showed no affinity to hordein, the prolamin of barley, whereas refolded tscFv did (Fig. 6c). For buckwheat neither anti-PT-gliadin-IgY nor refolded tscFv showed any neutralization capabilities (Fig. 6d). This comparative feasibility experiment demonstrated the desired biological activity of the refolded tscFv, which is why we analyzed this novel molecule also with flours of other grains.

#### Binding capabilities of the refolded tscFv

**Fig. 7:**
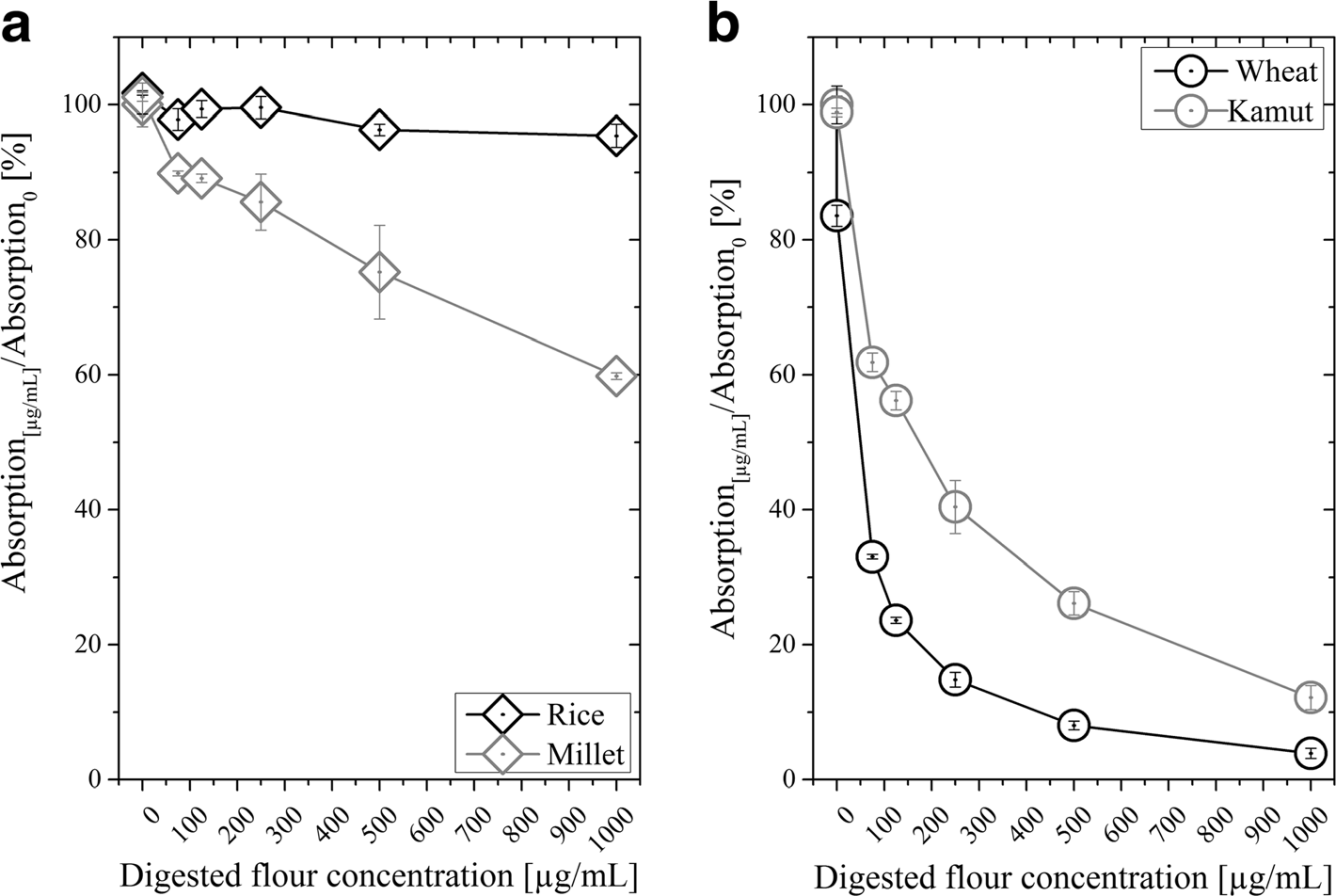
Competitive ELISA of refolded tscFv and flours considered as safe (**a**) as well as flours known to trigger CD (**b**). The ability of flours from different grains to replace refolded tscFv from immobilized PT-gliadin was tested. The tscFv was applied in a concentration of 8 μg/ml with flours in predefined total protein concentrations (0, 0.0075, 75, 125, 250, 500 and 1000 μg/mL). The relative signal in % is shown. 100% signal corresponds to the signal obtained with tscFv without any flour

We analyzed the refolded tscFv in more detail for its missing affinity towards digested flours, that are certified as safe, namely maize, soy, buckwheat, almond, millet and rice (exemplarily shown in Fig. 7a) as well as its binding capabilities for prolamins known to trigger CD, namely barley, rye, spelt, wheat and kamut (exemplarily shown in Fig. 7b).

As presented in Fig. 7a, the tscFv showed basically no activity with the flours of rice and millet. Slight responses observed for millet were due to the high concentration of digested flours, which led to a hindered interaction of immobilized PT-gliadin and tscFv. Also for the flours of other plants, which are basically prolamin-free, namely maize, soy, buckwheat and almond, we did not detect any biological activity. However, the tscFv bound to flours from grains containing prolamins, as exemplarily shown for wheat and kamut in Fig. 7b. For better comparability, we calculated IC50 values for these flours, which indicate the concentration of PT-gliadin or digested flour, where the respective signal of the ELISA was reduced by half (Table 3). Low values indicate high affinity of tscFv and vice versa. As shown in Table 3, the lowest value of 5.79 was found for the pure antigen PT-gliadin, followed by spelt and wheat. Since we found the desired biological activity of the novel tscFv, we concluded that it represents a highly interesting treatment option for patients suffering from CD, since it might be used as a medical device, which does not interact with the immune system.

**Table 3:** Results of non-linear regression of the values received from competitive ELISAs

	Sigmoidal, 4PL, X is log (concentration)			
	Applied grain	HillSlope	IC50 [μg grain/μg tscFv]	R square
prolamin containing grains	PT-gliadin	−1.26	5.79	0.998
	Wheat	−1.16	16.26	0.998
	Barley	−0.75	94.44	0.997
	Rye	−0.30	22.23	0.998
	Kamut	−0.63	32.60	0.998
	Spelt	−0.90	11.84	0.997

## Discussion

CD is a chronic disease involving the innate and adaptive immune system [1]. The immune system of genetically predisposed individuals responds to the dietary uptake of prolamin with inflammatory processes of the small intestine [3]. Hence, a strict livelong GFD has to be maintained and is currently the only option. However, a GFD is challenging because of hidden prolamins and costly dietary products, but also due to fear of prolamin exposure and hence possible social isolation [4, 33]. Thus, alternative and additional therapies are highly anticipated. In this study, we present a novel tscFv against various prolamins as a potential therapeutic support for patients with CD. The tscFv, selected from a chicken gene library, was recombinantly produced in *E.coli* as IBs. It is known that such molecules are difficult to express in *E. coli* in a soluble form [34]. We achieved an IB titer of 2.3 g per L cultivation broth, corresponding to 4.1 mg tscFv/g DCW/h induction time. This productivity is comparable to other biopharmaceuticals, such as Hirudin variant 1, where a specific productivity of 6.0 mg/g/h was achieved [35]. Even well-established processes, such as the production of insulin, only give a 3-times higher productivity of 14.2 mg/g/h [36].

We demonstrated that the tscFv IB itself shows biological activity. However, compared to the refolded tscFv at least 10-fold more tscFv IBs must be used to obtain a comparable biological effect. This circumstance clearly demands for the refolded product.

Renaturation of tscFv IBs, followed by ultra- and diafiltration, yielded 32% correctly folded target protein which represents a typical refolding yield in literature [37, 38]. During the IB process around 40% of product fragmented. However, we expect to further boost the refolding yield and reduce fragmentation by 1) buffer optimization; 2) determination of refolding kinetics and consequent adaptation of the process; 3) addition of stabilizers to reduce fragmentation (MS results indicated that the peptide linker was not stable during IB processing); and 4) changing the strategy from batch refolding by dilution to fed-batch refolding in the controlled environment of a refolding vessel.

When we investigated the binding capabilities of the tscFv with different flours, we found that lower concentrations of flours were capable to remove the standard polyclonal anti-PT-gliadin-IgY than refolded tscFv. This can be explained by the presence of product related impurities in the tscFv preparation (fragments) with lower binding affinity, which were confirmed by MS and HPLC analysis. Interestingly, anti-PT-gliadin-IgY showed no neutralizing effect with flour from barley. Only at high flour concentrations a reduction of the absorption signal was observed. However, this reduction is more likely explained by the high concentration of digested flower rather than the biological activity of anti-PT-gliadin-IgY. The tscFv not only shows a superior behavior towards the prolamins of barley compared to anti-PT-gliadin-IgY, but also compared to the scFv we examined in our previous study [11]. This higher binding affinity due to dimerization (and multimerization) is known in literature [12, 13]. Our binding study of tscFv with flours from different grains showed the desired outcome: tscFv bound to prolamin-containing flours, whereas no activity was detected with flours from grains, which are considered to be prolamin-free. We also performed an epitope mapping of the tscFv. We were able to identify the core epitope of the tscFv. The core epitope consists of an amino acid sequence containing almost exclusively prolines and glutamines - exactly those amino acids, which are problematic to digest in the gluten fraction and are contained in problematic prolamins. It also showed that the tscFv is binding to the 33-mer prolamin sequence, which is considered the most immune-toxic one, although with low affinity**.**

For a future application of this molecule we intend to deliver the tscFv to the intestine without getting destroyed by the hostile environment in the stomach. Packing the tscFv in micropellets coated with a gastric acid resistant film - traditionally by using shellac - is a suitable option for that purpose and has already proven to be extremely useful for two of our previous products (DAOsin® and FRUCTOsin®). The galenic formulation in micropellets has two advantages. First, some micropellets pass the stomach very fast (like liquids) because they are not retarded by the pylorus. This ensures that tscFv is instantly provided together with prolamin containing food. Secondly, the micropellets staying in the stomach are delivered gradually with the chyme - constantly supplying tscFv. Furthermore, in a first feasibility experiment we tested the stability of the tscFv in the presence of two prominent enzymes in the stomach – namely trypsin and chymotrypsin – and still found more than 50% of its initial biological activity after a 4 h incubation time (data not shown). In summary, we present a novel molecule, which can help patients suffering from CD. Our tscFv binds prolamins and can be used as a medical device. In vitro studies with Caco cell lines were promising and in vivo toxicity studies are currently ongoing.

## Conclusion

Here we present a novel tscFv as an interesting medical device to support patients suffering from celiac disease. We show the production of this molecule as insoluble protein aggregates in *E. coli*, called inclusion bodies, and the subsequent processing to obtain correctly folded and active product. Finally, we demonstrate the biological activity of this tscFv and compare it to a standard anti-PT-gliadin-IgY. Overall, we believe that the tscFv will be an important therapeutic support, leading to reduced dietary complications triggered by the consumption of prolamins for patients suffering from celiac disease.

## Additional file

Additional file 1: **Figure S1**. Block flow diagram of the workflow to generate the novel tandem single chain Fragment variable (tscFv) [11]. Red boxes show the immunization of the chicken, green boxes the identification and extraction of genes carrying the antigen binding site against peptic tryptic digested gliadin and blue boxes depict the simplified cloning strategy for the generation of the tscFv. (JPG 553 kb)

## Abbreviations

AB: Antibody
BM: Biomass
BSA: Bovine serum albumin
CD: Celiac disease
DCW: Dry cell weight
DSP: Downstream processing
ELISA: Enzyme-linked immunosorbent assay
ESI: Electrospray ionization
GFD: Gluten free diet
HLA: Human leukocyte antigen
IB: Inclusion body
IC50: Half maximal inhibitory concentration
IEL: Intraepithelial lymphocyte
IFN: Interferon
IL: Interleukin
IPTG: Isopropyl β-D-1-thiogalactopyranoside
PT-gliadin: Peptic tryptic digested gliadin
scFv: Single chain Fragment variable
TBST: Tris buffered saline with 0.05% Tween 20
TG2: Tissue transglutaminase2
TMB: 3,3′,5,5′-tetramethylbenzidine
TNF: Tumor necrosis factor
tscFv: Tandem single chain Fragment variable
WW: Wet weight
