# Supplementary figures and images for: The production of a recombinant tandem single chain fragment variable capable of binding prolamins triggering celiac disease
Source: BMC Biotechnol. 2018 May 29;18:30. doi: 10.1186/s12896-018-0443-0 (PMC5975707; doi:10.1186/s12896-018-0443-0)

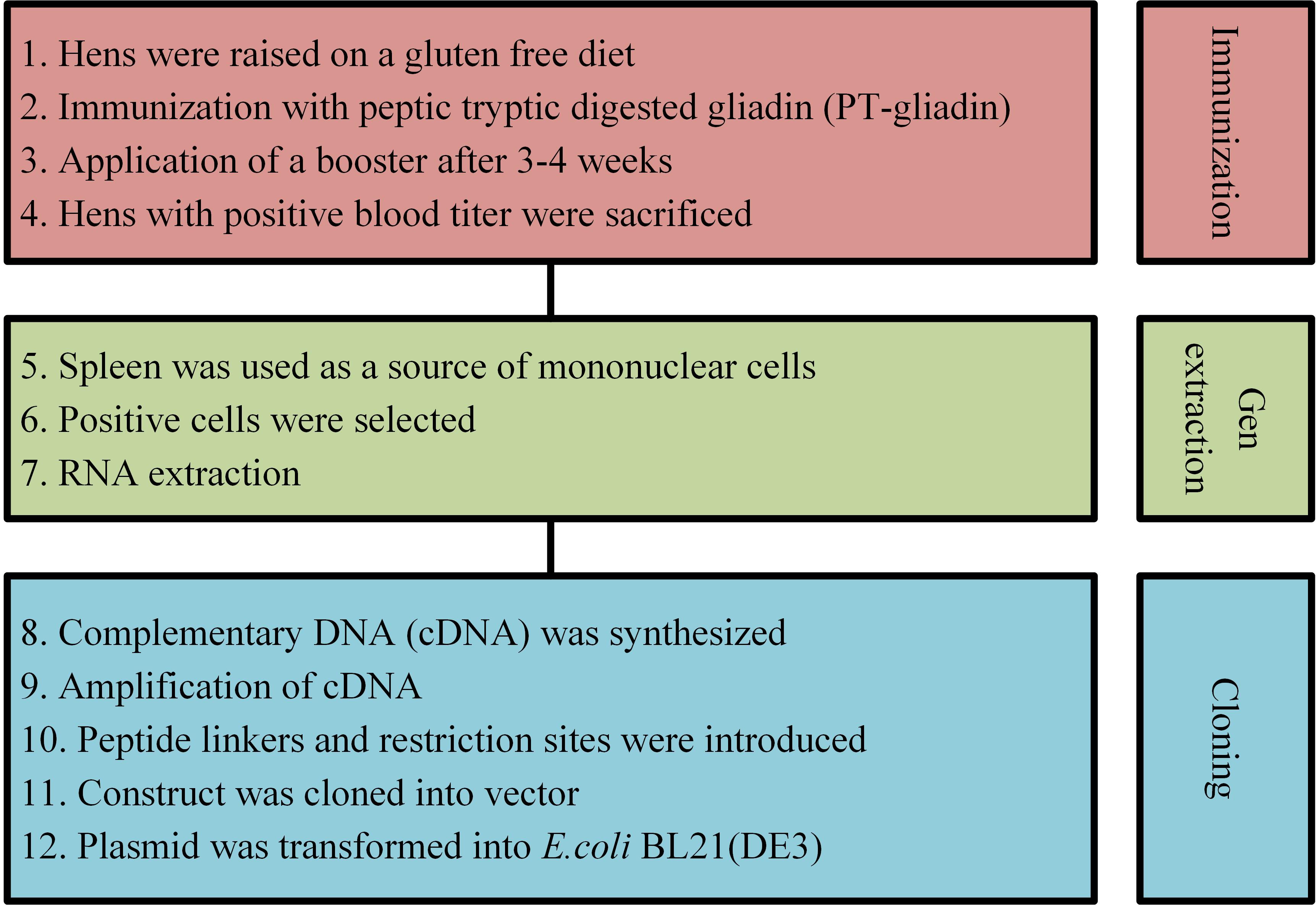

Supplement: Supplementary file 1 — Figure S1. Block flow diagram of the workflow to generate the novel tandem single chain Fragment variable (tscFv) [11]. Red boxes show the immunization of the chicken, green boxes the identification and extraction of genes carrying the antigen binding site against peptic tryptic digested gliadin and blue boxes depict the simplified cloning strategy for the generation of the tscFv. (JPG 553 kb) [file 12896_2018_443_MOESM1_ESM.jpg]
